# Microstructure-informed brain tissue classification using clustering of quantitative MRI measures

**DOI:** 10.1162/imag_a_00526

**Published:** 2025-04-03

**Authors:** Sharada Balaji, Marek Obajtek, Irene M. Vavasour, Adam Dvorak, Guillaume Gilbert, Poljanka Johnson, Roger Tam, Cornelia Laule, David K.B. Li, Anthony Traboulsee, Alex MacKay, Shannon H. Kolind

**Affiliations:** Physics and Astronomy, University of British Columbia, Vancouver, BC, Canada; Radiology, University of British Columbia, Vancouver, BC, Canada; International Collaboration on Repair Discoveries, University of British Columbia, Vancouver, BC, Canada; MR Clinical Science, Philips Canada, Missisauga, ON, Canada; Medicine (Neurology), University of British Columbia, Vancouver, BC, Canada; Pathology & Laboratory Medicine, University of British Columbia, Vancouver, BC, Canada

**Keywords:** myelin water imaging, tensor-valued diffusion, clustering, tissue microstructure, tissue classification

## Abstract

Traditional tissue classification approaches in vivo use voxel intensities from conventional clinical magnetic resonance (MR) images for segmentation, which does not incorporate information about specific aspects of microstructure. With the Clustering for Anatomical Quantification and Evaluation (CAQE) framework, quantitative MRI measures can be used to classify tissue based only on microstructural features with no spatial enforcement, and pathological changes in disease can be evaluated. In this study, maps of whole-brain myelin water fraction, microscopic fractional anisotropy, and tissue heterogeneity were used to classify brain tissue in 25 healthy participants. CAQE was then applied to 25 participants with multiple sclerosis (MS), where tissue classifications indicated areas of increased demyelination and axonal injury in white matter compared with a healthy average tissue classification. Severity scores were derived from tissue classifications to quantify diffuse white matter damage, and correlated significantly with cognitive ability in MS. The CAQE framework can be adapted for other applications and extended to use different quantitative MRI measures.

## 1 Introduction

Magnetic resonance imaging (MRI) is commonly used to differentiate between broad categories of central nervous system (CNS) tissue, such as distinguishing white matter from grey matter, or areas of focal damage such as tumor, stroke, or lesion from healthy tissue. However, the ability to categorise CNS tissue with more detail and biological specificity would be of great value for tasks ranging from segmenting different thalamic nuclei to investigating subtle, diffuse damage not easily identified using conventional MRI. For example, multiple sclerosis (MS), a chronic inflammatory disease of the CNS, involves diverse pathological changes that can occur throughout the course of the disease in different parts of the brain and spinal cord (Popescu et al., 2013;Reich et al., 2018). Focal lesions, the hallmark of MS, are visible using conventional clinical MRI scans, and the size and number of lesions (lesion load), as well as global brain atrophy correlate only weakly with clinical scores and progression (Reich et al., 2018). Widespread damage occurs in the normal-appearing white matter (NAWM) outside of these lesions, including inflammation, edema, demyelination, axonal damage or loss, and changes to iron concentration. However, these subtle NAWM changes are not easily visible or distinguishable with clinical MRI. Additionally, microscopic pathological features of lesions can be heterogeneous, which is also not discernable on conventional imaging.

Quantitative MRI techniques can provide measures for different aspects of tissue microstructure such as myelin content or axonal integrity. Combining quantitative MRI measures that inform on specific features may provide a more comprehensive view of CNS microstructure, quantify the severity of tissue damage and its relationship to disability, and allow for improved understanding of the various microstructural changes that may drive progression in neurodegenerative diseases such as MS.

One such quantitative MRI technique, myelin water imaging (MWI), provides the myelin water fraction (MWF) (A. Mackay et al., 1994), a histopathologically validated biomarker for myelin content in the CNS (Laule et al., 2006,^2008^). MWI quantifies the T_2_relaxation times in different water compartments of CNS tissue, and typically works by acquiring a T_2_decay curve, which is then decomposed using a non-negative least squares algorithm into a T_2_distribution (A. Mackay et al., 1994). The signal with T_2_< 40 ms arises from water trapped within myelin lipid bilayers, and the ratio of this short-T_2_signal to the total water signal provides the MWF for every voxel in the image. The MWF has been known to change over the healthy lifespan (Dvorak et al., 2021;Faizy et al., 2020;Morris et al., 2020) and has been used to study a variety of diseases such as Huntington’s Disease (Casella et al., 2021), leukodystrophies (Laule et al., 2018;Van De Stadt et al., 2021), and MS (Kolind et al., 2012). In MS, the MWF shows areas of focal demyelination in lesions, as well as generally reduced values in the NAWM (A. L. MacKay & Laule, 2016). MWI acquisition techniques are approaching clinical feasibility, with scan times as low as 5 minutes for whole brain coverage using the recently introduced CALIPR framework (Dvorak et al., 2023), and a new 3D spatial correlation-based analysis technique (Kumar et al., 2018) providing robust MWF maps that show pathology with improved sensitivity.

A different branch of quantitative MRI, diffusion tensor imaging, uses the diffusion of water molecules in tissue to infer details about the underlying tissue microstructure by calculating a diffusion tensor for each voxel in the image. A new variant of diffusion imaging, tensor-valued diffusion imaging, aims to resolve heterogeneous tissue environments in the acquisition itself (Topgaard, 2017). Each voxel is assumed to be composed of multiple tissue micro-environments with distinct diffusion tensors that can be calculated. The associated signal model and framework (q-space trajectory imaging, QTI (Westin et al., 2016)) provides methods to calculate voxel-level means and variances of the diffusion tensors of these micro-environments. The microscopic fractional anisotropy (µFA) can be derived using the QTI framework, and is a measure of the anisotropy within a voxel without the confound of orientation dispersion, and thus provides a marker for axonal integrity. In MS, µFA has previously shown reduced values in NAWM compared with healthy WM (Andersen et al., 2020;Yang et al., 2018). Another measure from the QTI framework shows the variance of mean diffusivities within the voxel and can be considered a marker of tissue heterogeneity, C_MD_. This has previously been linked to cellularity in tumor (Nilsson et al., 2020). The tensor-valued diffusion approach has previously been used in studies of MS (Andersen et al., 2020;Yang et al., 2018), tumor (Nilsson et al., 2020), and cancer (Nilsson et al., 2021), with validation in phantoms (Nilsson, Larsson, et al., 2018) and pathology (Brabec et al., 2022).

MWF, µFA, and C_MD_can together paint a more complete picture of brain microstructure than each measure alone as they provide information about different aspects of tissue microstructure, and more insight into biological processes than conventional, qualitative MRI scans. However, assessing three quantitative MRI maps to determine tissue characteristics is a time-consuming task that could be made more efficient by viewing a combination of all the data. MWF, µFA, and C_MD_could be used to classify tissue, with the classification presented as a single visual map. Such a microstructure-based tissue classification would provide more detailed separation and information about tissue types than traditional tissue segmentation algorithms, which work based on intensities of qualitative MRI images and typically separate tissue into grey matter, white matter, and cerebrospinal fluid (CSF).

In this study we present a flexible framework, Clustering for Anatomical Quantification and Evaluation (CAQE), for clustering and classifying brain tissue based only on microstructural features without spatial input. This was developed and demonstrated in three parts: (a) we developed a model to classify tissue based on clustering MWF, µFA, and C_MD_data from 25 healthy controls (HC); (b) we applied the model to 25 MS datasets to determine where and how tissue microstructure changes in MS; and (c) we derived an overall score to quantify tissue changes in MS, and investigated relationships between these tissue classifications and clinical and cognitive ability. The CAQE framework is flexible, can provide a characteristic multidimensional pattern of microstructural features for each tissue class regardless of its location, and visualise multifaceted changes to tissue microstructure in the form of a single map.

## 2 Methods

### 2.1 Population and MRI acquisitions

MRI data were acquired in 25 HCs, 11 participants with relapsing-remitting MS (RRMS), and 14 participants with progressive MS (ProgMS). Expanded Disability Status Scale (EDSS) and Oral Symbol Digit Modalities Test (SDMT) scores were obtained for MS participants.Table 1describes the characteristics of each group. All volunteers provided informed, written consent (University of British Columbia Clinical Research Ethics Board, Number H17-00866). This study followed the Strengthening the Reporting of Observational Studies in Epidemiology (STROBE) guidelines.

**Table 1.** Demographic characteristics.

	Healthy controls (HC, n = 25)	Relapsing-remitting MS (RRMS, n = 11)	Progressive MS (ProgMS, n = 14)
Age	46 ± 15 y Range = 23–70 y	50 ± 12 y Range = 31–65 y	64 ± 5 y Range = 53–70 y
Sex	11M, 14F	3M, 8F	4M, 10F
Expanded disability status scale (EDSS)	-	Median = 2.5 (2–3.5)	Median = 3.5 (1.5–6.5)
Oral symbol digit modalities test (SDMT)	-	Mean = 66 (56–86) [n = 10]	Mean = 52 (39–70) [n = 14]

Details of each group and corresponding EDSS and SDMT scores. For one RRMS subject, SDMT scores were not available.

Imaging was performed at 3T (Philips Ingenia Elition X, Philips Healthcare, Best, the Netherlands) using a 32-channel receive-only head coil. A 3D T_1_-weighted sequence was acquired (shot interval = 2400 ms, TR = 7.7 ms, TE = 3.7 ms, TI = 950 ms, FOV = 256 x 180 x 256 mm^3^(APxRLxFH), resolution = 1 x 1 x 1 mm^3^, 3.5 minutes) for image registration and to create a structural template. For lesion visualisation in all MS participants, 3D T_2_-FLAIR (TR = 8000 ms, TE = 310 ms, TI = 2400 ms, FOV = 256 x 181 x 256 mm^3^, 5 minutes) images were also acquired at resolution 1 x 1 x 1 mm^3^, reconstructed to 0.67 x 0.67 x 0.67 mm^3^.

MWI data were collected using the CALIPR sequence (Dvorak et al., 2023) (56 echoes, TR = 1252 ms, ΔTE = 6 ms, FOV = 240 x 200 x 100 mm^3^, acquired at 1.7 x 1.7 x 1.7 mm^3^, reconstructed to 1 x 1 x 1 mm^3^, undersampling acceleration factor = 23.9, 7.5 minutes). Tensor-valued diffusion acquisitions (Sjölund et al., 2015;Szczepankiewicz et al., 2021) (TR = 5200 ms, TE = 129 ms, δ_pause_= 17.5 ms, FOV = 240 x 240 x 144 mm^3^, resolution = 3 x 3 x 3 mm^3^, multiband SENSE factor = 2, SENSE factor = 1.9, half scan factor = 0.74) with spherical tensor encoding (*STE*, 33 rotations, 3 minutes), planar tensor encoding (*PTE*, 32 rotations, 3 minutes), and linear tensor encoding (*LTE*, 59 directions, 5 minutes) were performed. Reverse phase-encoded images without diffusion weighting were acquired in 44 of 50 participants for distortion correction.

### 2.2 Data processing

For each individual, MWF maps were generated from the CALIPR data using a spatial correlation method with two spatial iterations (Kumar et al., 2018). Diffusion data were pre-processed with tools from FSL and MRTrix3 (Tournier et al., 2019) for Gibbs ringing removal (Kellner et al., 2016), denoising (Veraart et al., 2016), and susceptibility distortion correction (Andersson et al., 2003). Where reverse phase-encoded data were not available, Synb0-DisCo (Schilling et al., 2019) was used to generate undistorted images. Motion and eddy current correction was performed by extrapolating reference volumes (Nilsson et al., 2015) through an open-source Matlab library (Nilsson, Szczepankiewicz, et al., 2018) using ElastiX (Klein et al., 2010). Maps of µFA and C_MD_were obtained through the QTI+ framework (Boito et al., 2022;Herberthson et al., 2021). Each participant’s µFA and C_MD_maps were registered to their MWF map prior to clustering by registering the b = 0 image of diffusion to the 1^st^echo of MWI using FSL’s epi_reg and applying the resulting transform to µFA and C_MD_maps. All registrations were manually checked. CSF was masked out to remove it as a confound in the clustering process using ANTs Atropos (Avants, Tustison, Wu, et al., 2011). In HCs, this was done on T_1_-weighted images to generate CSF and tissue (WM+GM) masks that were then warped to MWI space. In MS, Atropos was run on both FLAIR and T_1_-weighted images, and tissue segmentations were warped to MWI space and combined to generate an overall CSF and tissue (WM+GM) mask that included lesions.

All HCs’ T_1_-weighted images were consolidated into a healthy template using ANTs’ multivariateTemplateConstruction (Avants, Tustison, Song, et al., 2011), and atlases of MWF, µFA, and C_MD_were created by registering to this template with ANTs’ symmetric diffeomorphic registration (Avants, Tustison, Song, et al., 2011) for future comparisons of individual subjects to a healthy average.

### 2.3 Clustering and classification

Principal component analysis (PCA) was used to determine the relationship between the measures, MWF, µFA, and C_MD_. PCA was performed on a single pool of whole-brain data (CSF-masked) from 25 HCs (a matrix of dimensions ~23 million x 3), and explained variance ratios were calculated. A correlation matrix of the three measures was also calculated.

Data from the 25 HCs were used to create a tissue classification model. The 25 sets were randomly divided into a training (n = 20) and testing (n = 5) set. Data from training subjects were loaded into a single pool where individual datapoints were a specific combination of MWF, µFA, and C_MD_, with no spatial input involved. This pool was scaled using a standard Gaussian scaler and clustered using scikit-fuzzy’s (Warner et al., 2019) Fuzzy C-Means algorithm (Ross, 2010) to label datapoints. Each test subject’s data were then classified based on the clustering using scikitlearn’s (Pedregosa et al., 2011) K-Nearest Neighbours algorithm. The “best” number of clusters was determined using Calinski-Harabasz (Calinski & Harabasz, 1974) and Davies-Bouldin (Davies & Bouldin, 1979) scores. The 25 subjects were then split into a different set of training (20) and testing (5) data and the clustering/classification process was repeated. This was repeated 5 times with different sets of training and testing data, such that by the end, each HC was classified based on 20 other HCs. Rotating through groups of testing/training sets allowed for determination of the variability in tissue classification; this was a homegrown variant of the cross-validation paradigm to ensure stability in clustering results (Ullmann et al., 2022). Once the best number of clusters was decided, a tissue classification model was built based on all 25 HCs.

**Fig. 1.**
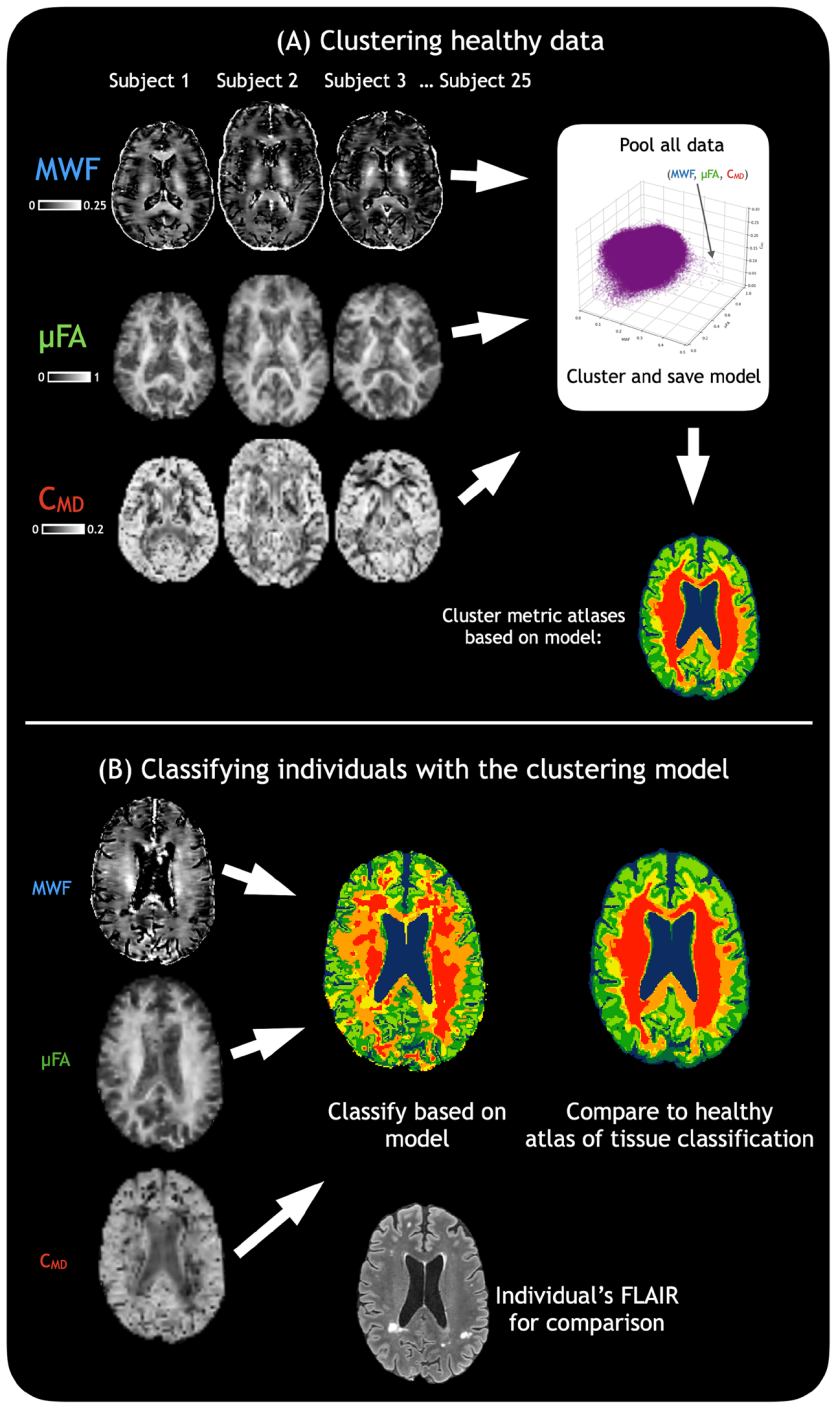
Flow of data in the CAQE framework. (A) Represents the clustering process performed on all healthy data, which only needs to be performed once for a given set of metrics after the optimal number of clusters is found. (B) Represents the classification process for any individual outside of the training set where the same MRI metrics have been acquired, whether healthy or with a neurological condition (shown here is an example of MS). This tissue classification can be compared with a healthy average to judge areas of tissue damage (here for example, periventricular lesions are classified as yellow). Cluster colours for this workflow figure are illustrative only.

MWF, µFA, and C_MD_atlases were classified based on training with all 25 HCs, to serve as a population-averaged tissue classification for comparison with individual subjects. This is referred to here as the “atlas of tissue classification.” Every single MS participant’s data were then classified based on the model of all 25 HCs and compared with the atlas of tissue classification.Figure 1shows the flow of data in this framework once the optimal cluster number has been decided.

Clusters were organised by order of mean MWF for ease of interpretation in this work, with #1 having the highest mean MWF. For each subject (HC and MS), the normalised size of each cluster was calculated as

$$Normalised\;cluster\;size=\;\frac{\#\;of\;voxels\;in\;cluster}{Total\;voxels\;in\;subject^{\prime }s\;brain}$$

### 2.4 Severity scores

The atlas of tissue classification was warped to each individual MS participant’s MWI space, so that each participant’s tissue classification could be directly compared with the healthy atlas. “Difference maps” for each MS participant were made by comparing regions on the healthy atlas with equivalent locations in the MS participant and marking where clusters denoting white matter (clusters 1–3) in the healthy atlas differed in MS. The magnitude on the difference maps represents, for a given voxel, the difference in cluster number between the atlas and the MS participant; for example, a cluster difference of 2 denotes a voxel labeled #1 on the atlas being classified as #3 in the MS participant instead. Opposite cluster number changes (e.g., where a voxel labelled #2 on the atlas is labelled #1 in the MS participant) were not considered, as they would imply higher myelin content than the healthy atlas, which would be unusual in MS unless affected by other confounds. However, for completeness, these opposite cluster number changes were evaluated as a fraction of total voxels in the brain.

From these difference maps, a severity score was determined for each MS participant by scaling only the cluster difference of 1.

$$Severity\;score=\left(\frac{\#\;of\;voxels\;showing\;cluster\;difference\;of\;1}{Total\;voxels\;in\;subject^{\prime }s\;brain}*1000\right)-50.$$

These severity scores were not normalised within the current study population to facilitate comparison with future studies. Scores derived from other cluster differences (such as 2, 3, and higher) were also calculated for completeness as they may also be meaningful.

### 2.5 Statistical tests

Normalised cluster sizes were compared at a group level using one-way ANOVA between HC, RRMS, and ProgMS. Cluster sizes that showed significant (P < 0.05) differences were further compared with Tukey’s Honestly Significant Difference (HSD) pairwise group comparisons to determine which specific groups showed differences. Severity scores of MS participants were compared between RRMS and ProgMS using an unpaired two-sided Welch’s t-test; this test was chosen after manually checking that the variances and population sizes of ProgMS and RRMS severity scores did not match.

The relationship between severity scores and SDMT scores was examined using Spearman’s correlations in all MS, and in RRMS and ProgMS separately; the Spearman’s test was chosen since the data were not assumed to be linearly related or normally distributed. Scores derived from 2-cluster, 3-cluster, and higher differences between the atlas of tissue classification and each MS participant were also compared with SDMT and EDSS scores using Spearman’s correlations.

## 3 Results

### 3.1 CAQE framework

A clustering model was created based on HC data to classify tissue based on MWF, µFA, and C_MD_maps as detailed inSection 2, and the model was then used to classify MS tissue. The best number of tissue clusters was determined through a cross-validation method of training and testing, which involved training on data from 20 HCs and testing on data from the remaining 5 HCs to determine the optimal number of tissue clusters, and then rotating between the data that were trained on and tested. Using metrics that were known to provide largely non-overlapping information was necessary for good performance of the clustering algorithm, to allow for better cluster separation. The metrics (MWF, µFA, and C_MD_), which inform on myelin content, tissue anisotropy, and tissue heterogeneity, indeed did not significantly overlap as shown by the PCA results inFigure 2. It was determined that all of the metrics play a non-negligible role in the data, as PC1 (which explains 55% of the variation in the data) and PC3 (which explains 13% of variation in the data) are dominated by µFA and C_MD_, and PC2 (which explains 32% of the variation) is dominated by MWF.

**Fig. 2.**
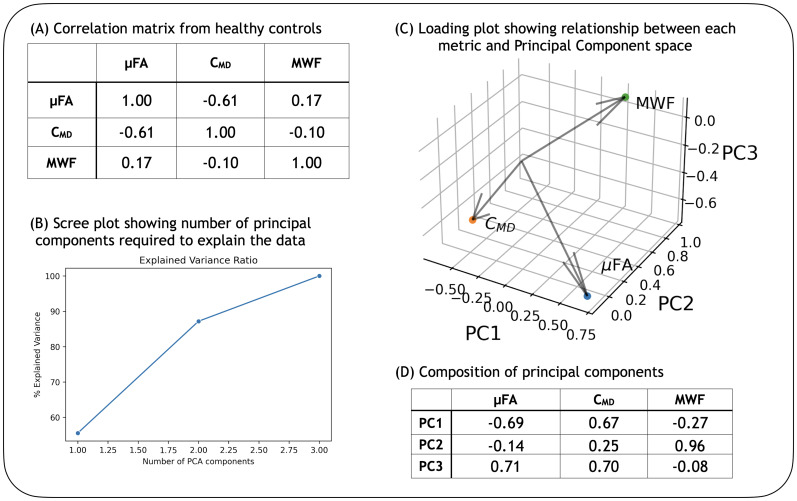
Results from PCA. Results from PCA performed on the three metrics to show their non-overlapping nature. (A) Shows the correlation matrix between the metrics, with µFA and C_MD_showing a moderate relationship and no strong relationship between MWF and µFA or C_MD_. (B) Shows that using PCA for dimensionality reduction still results in three components being required to explain the data, implying that no metric can simply be dropped. (C) Shows the projection of each of the original metrics onto the new PCA axes. (D) Shows the role that each metric plays in each principal component: PC1 (which explains 55% of the variation in the data) and PC3 (which explains 13% of variation in the data) are dominated by µFA and C_MD_, while PC2 (which explains 32% of the variation) is dominated by MWF. Therefore, none of the metrics play a non-negligible role in the data, and provide different information. Measures shown here were calculated from whole brain in 25 HCs, excluding CSF.

A clustering model was created based on all 25 HCs, and the full 25-subject model was also applied to some of the healthy control datasets to ensure that the clustering was consistent even with more training datapoints, shown inFigure 3.

**Fig. 3.**
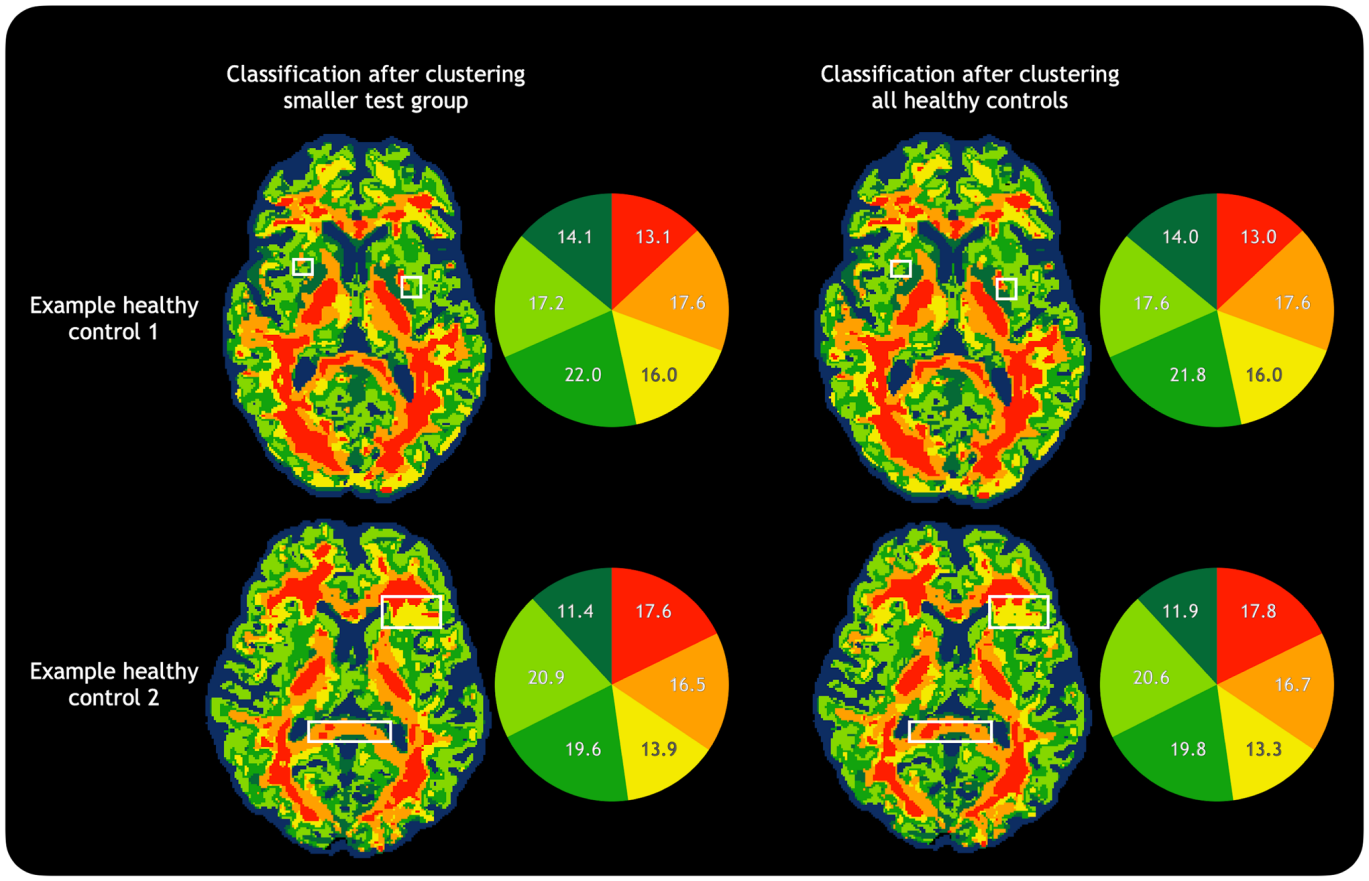
Comparison of different training sets. Comparison of tissue classification using a smaller training set (20 subjects) and the full HC population (25 subjects) for clustering in two example HCs. The training set made only minor changes such as the ones highlighted in white boxes, keeping the relative normalised cluster sizes largely consistent (pie charts). For this reason, when classifying HCs, the smaller training groups were used; this also prevented the pitfalls of testing members of the training dataset, although both methods resulted in similar tissue classifications and subsequent metrics.

The clustering model was used to classify “atlases” of MWF, µFA, and C_MD_to create an atlas of tissue classification, to act as a target for comparison for individual subjects showing pathology. Individual subjects, including those with pathology, can be classified using the clustering model and compared with the atlas.

### 3.2 Cluster interpretations in healthy controls

With the CAQE method of classifying tissue based on microstructural features alone, detailed classifications within white and grey matter were obtained. White matter tended to be classified into one of three clusters, grey matter into one of two clusters (including some overlap with sub-cortical white matter), and iron-rich deep grey matter was generally assigned as a separate cluster. CSF was not included in the tissue classification. It is important to note that voxels were assigned to a given cluster regardless of anatomical location, based only on the microstructural features as detected using the MRI metrics, and reference to each cluster as a particular tissue type is based on where these voxels tended to be and the microstructural properties associated with the MRI metrics for that cluster.

**Fig. 4.**
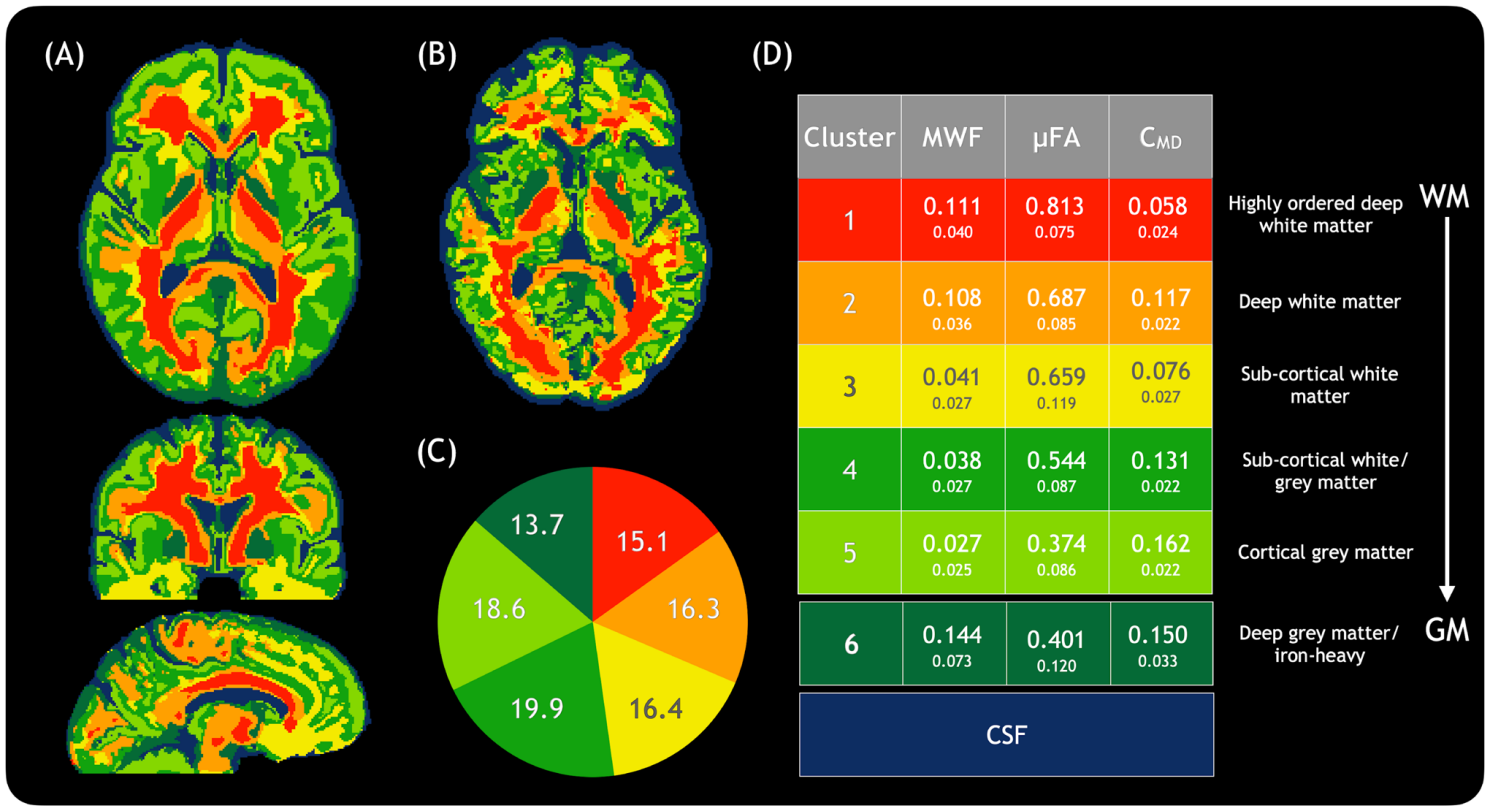
A representation of tissue classifications in healthy tissue. (A) Shows the tissue classification of the healthy average brain, based on 25 healthy individuals, which can be used as a comparison tool, while (B) shows an example of a single healthy individual; the same patterns in tissue classifications can be seen when compared with the healthy average. (C) Shows the relative sizes of different clusters across all healthy subjects, and (D) shows the metric values within each cluster (mean on top, standard deviation below). For example, Cluster #1, prevalent in areas of highly coherent deep white matter, shows high myelin content, high anisotropy, and low heterogeneity, while Cluster #5, present in cortical regions, shows low myelin content, low anisotropy, and high heterogeneity. CSF was not included in the tissue classification. The areas generally assigned to each cluster are indicated on the far right, with areas most often found in white matter (WM) towards the top and grey matter (GM) towards the bottom.

Figure 4shows (A) a representative slice from the atlas of tissue classification, (B) an example slice from a HC, (C) average normalised cluster sizes across all HCs, and (D) the values of measures that define each cluster. Clusters can be interpreted based on known features of brain microstructure in the areas usually assigned to that cluster, and the interpretation of the MRI metrics within that cluster. Cluster #1, a region of high myelin content, high anisotropy, and low heterogeneity, is present in areas of highly coherent deep white matter. Anatomically, Cluster #2 is also present in deep white matter, but has higher heterogeneity and lower anisotropy than Cluster #1, indicating tissue with a variety of fiber sizes. Cluster #3 anatomically corresponds to sub-cortical white matter and has lower myelin content and lower heterogeneity than Cluster #2, which suggests thinner myelin sheaths on axons with relatively uniform packing. Cluster #4 occupies a mixture of sub-cortical white and grey matter, including the border between the two, with relatively low myelin content, low anisotropy, and high heterogeneity. Cluster #5 differs from Cluster #4 with even lower anisotropy and higher heterogeneity, and corresponds to cortical grey matter. Finally, Cluster #6 was a seemingly contradictory cluster with apparently high myelin content, low anisotropy, and high heterogeneity. However, it is known that the anatomical regions which correspond with Cluster #6 (e.g. the globus pallidus) have high iron concentration, which is known to shorten the T_2_relaxation time and artificially inflate the MWF (Alonso-Ortiz et al., 2015). Therefore, Cluster #6 likely represents regions of high iron concentration in deep grey matter.

### 3.3 Clusters in MS

**Fig. 5.**
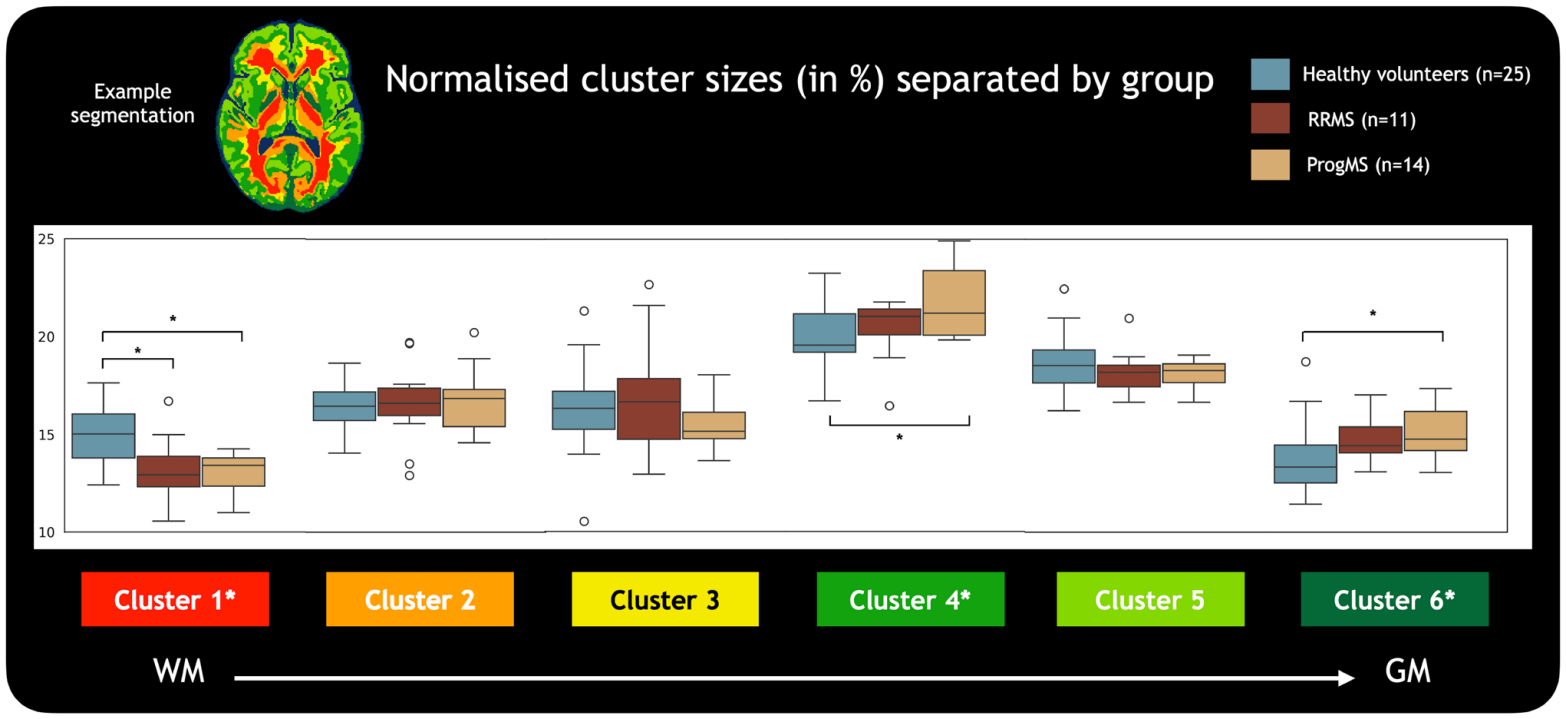
Comparison of normalised cluster sizes between different groups. The proportion of voxels assigned to Cluster #1 (with characteristics of highly coherent white matter) was significantly different between healthy controls and participants with MS, while the proportion assigned to Cluster #6 (with characteristics of iron-rich tissue) and Cluster #4 (with characteristics of sub-cortical white and grey matter) was also significantly different between healthy controls and ProgMS. (*) indicates significant differences (P < 0.05).

Normalised tissue cluster sizes were compared between MS and HCs at a group level, separated as HC, RRMS, and ProgMS (Fig. 5). Relative to HC, MS showed an overall decrease in the proportion of voxels assigned to Cluster #1 (with characteristics of highly coherent deep white matter, one-way ANOVA: F(2,22) = 11, P = 9.5E-6), increase in the proportion of voxels assigned to Cluster #4 (with characteristics of sub-cortical grey/white matter, F(2,22) = 4.89, P = 0.012), and increase in the proportion of voxels assigned to Cluster #6 (with characteristics of iron-rich tissue, F(2,22) = 4.53, P = 0.016). Further post hoc tests using Tukey’s HSD pairwise group comparisons for these clusters showed a significant difference in the proportion of voxels assigned to Cluster #1 between HC and both RRMS (P = 0.002, 95% CI = [0.006, 0.032]) and ProgMS (P < 0.001, 95% CI = [0.008,0.032]), Cluster #4 between HC and ProgMS (P = 0.009, 95% CI = [-0.031,-0.004]), and Cluster #6 between HC and ProgMS (P = 0.022, 95% CI = [-0.025, -0.002]).

**Fig. 6.**
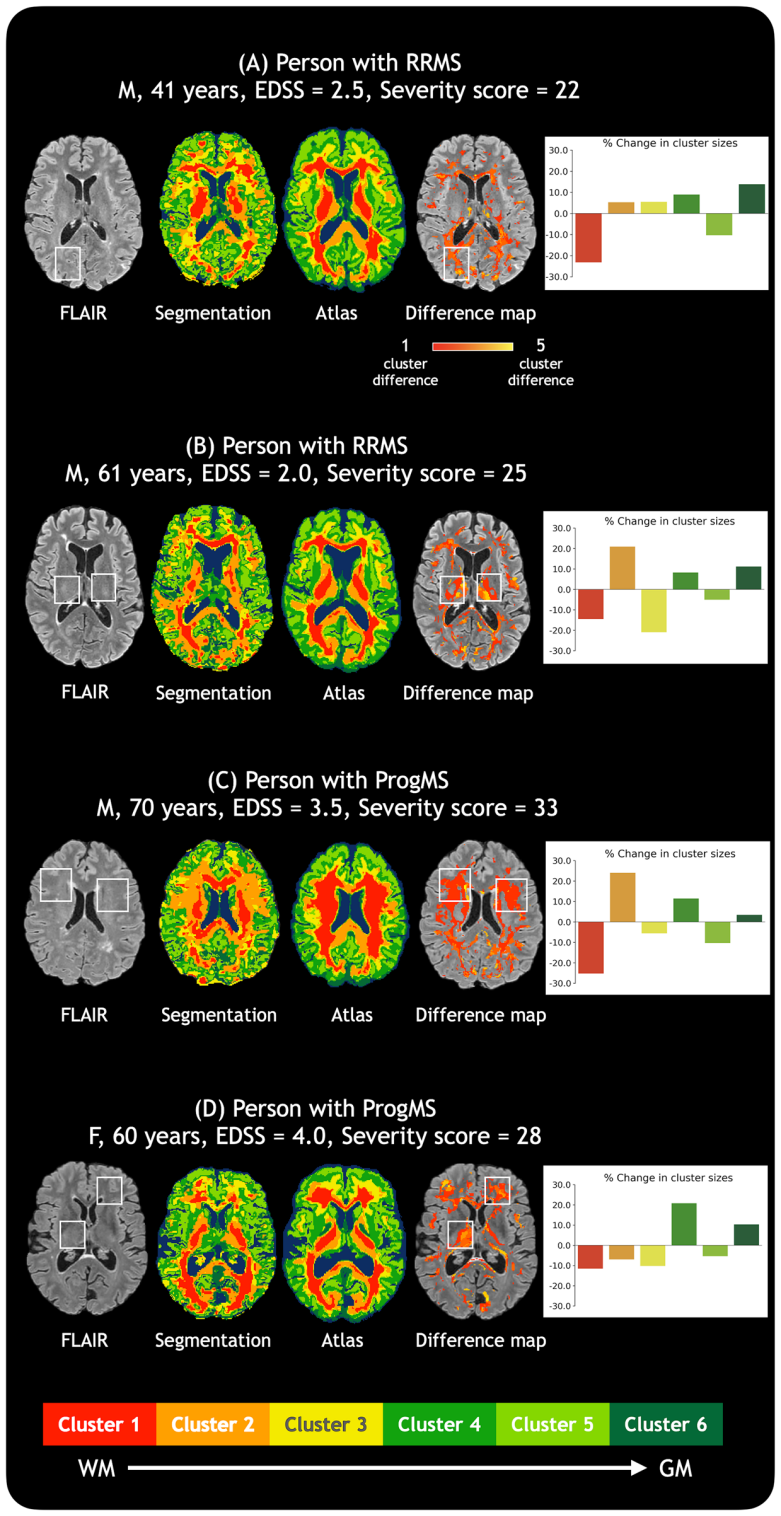
Examples of MS tissue classification. Four different MS participants (A–D) with varying MS phenotypes, EDSS scores, and severity scores are shown. The associated bar charts show the percent difference between each subject’s normalised cluster sizes and the mean normalised cluster sizes from the healthy population, with the colour bar at the bottom showing cluster number and colour associations. Difference maps indicate regions where the atlas and the MS participant differ in white matter classifications. White boxes on each participant’s FLAIR show example regions of abnormality in the participant compared with the healthy brain atlas that is not clearly visible on the FLAIR image itself. Lesions are generally classified as Cluster #3 (representing demyelination and axonal damage in MS), Cluster #4 (representing demyelination, axonal loss, and increased heterogeneity in MS), or both. Severity scores were determined from the proportion of voxels with a cluster difference of 1.

Figure 6shows four representative examples of MS compared with the atlas of tissue classification, with white boxes identifying abnormal regions compared with the atlas which are generally not visible on FLAIR. The difference maps show where the MS participant’s white matter tissue classification varied from the atlas. For example, inFigure 6(A), the white box marks out a region that is classified as Cluster #1 in the healthy atlas but was classified as Cluster #3 in the RRMS participant. InFigure 6(B), white boxes show regions that are Clusters #1–2 in the healthy atlas but are classified as Clusters #4–6 in the RRMS participant.Figure 6(C)shows an example where Cluster #1 in the healthy atlas is classified as #2 in the ProgMS participant, whileFigure 6(D)shows an example where Clusters #1–2 in the healthy atlas are classified as #3 and #4 in the ProgMS participant. The associated bar charts for each participant show the percent change in the size of each cluster for the individual compared with all HCs. Across all these examples, there was a general trend towards Cluster #1 (with characteristics of highly coherent deep white matter) being classified as clusters with lower myelin content and anisotropy. Higher proportions of voxels assigned to Cluster #4 and Cluster #6 are also present in all examples, which is corroborated byFigure 5at a group level.

### 3.4 Severity scores

**Fig. 7.**
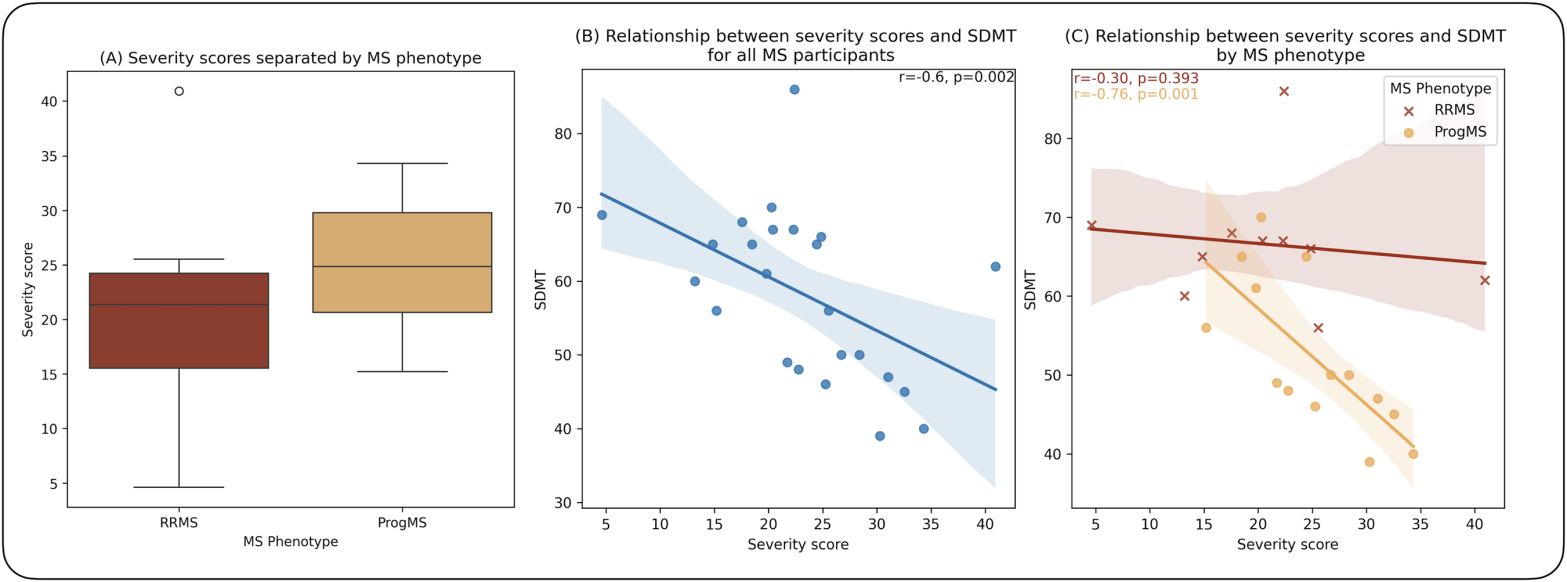
Relationships with severity scores. (A) Shows the difference between MS phenotype severity scores, with ProgMS generally presenting higher values, although not significantly (t(23) = -1.35, P = 0.19). (B) Shows the relationship between severity scores and SDMT scores for all MS participants (Spearman’s r = -0.60, P = 0.002). (C) Shows the relationship between severity scores and SDMT scores separated by MS phenotype, with a stronger correlation present in ProgMS (Spearman’s r = -0.76, P = 0.001). The RRMS outlier (with severity score > 40) does not affect the relationship with ProgMS in (C), but does impact the overall MS relationships presented in (B).

Severity scores were calculated by comparing tissue classifications in MS with the HC atlas of tissue classification, and reflected diffuse changes to white matter integrity through demyelination and axonal damage. Although a Welch’s two-sided t-test showed no significant difference between the severity scores of RRMS and ProgMS (t(23) = -1.35, P = 0.19), the two sets of scores were visibly different (Fig. 7(A)).

One person with RRMS did not have an SDMT score available and was, therefore, excluded from the correlation analysis with severity scores. Severity scores were significantly correlated with SDMT scores across all MS (n = 24, Spearman’s r = -0.60, P = 0.002,Fig. 7(B)), and the relationship was driven by ProgMS (n = 14, Spearman’s r = -0.76, P = 0.001,Fig. 7(C)). No significant relationships were found between severity scores and EDSS.

## 4 Discussion

CAQE provides a framework for combining data from multiple advanced MRI techniques to classify healthy and pathological brain tissue in vivo in a single map based only on microstructural features. MS pathology in lesions and NAWM is heterogeneous within and between people, and can include de/re-myelination, inflammation, and edema, axonal injury and loss, and/or increases in iron concentration (Lassmann, 2003,^2018^;Reich et al., 2018). By classifying MS brain tissue based on multiple quantitative MRI metrics, regions of brain abnormalities that are not evident on conventional images can be observed, with the added benefit of being able to interpret such changes in terms of tissue microstructure from a single map.

### 4.1 CAQE approach

Due to the nature of this unsupervised approach, the number of clusters is unknown a priori. The “best” number of clusters was determined using test scores to quantify aspects of between-cluster distances and cluster dispersion, as well as judgement based on visual interpretation. The best number of clusters (six) for the healthy population showed consistent patterns of tissue classification across subjects regardless of the training dataset.Figure S1shows these clustering scores from 5 test subjects trained with 20 subjects. As an alternative to the six clusters presented in the paper,Figure S2shows four (the other “best” number of clusters) in one healthy control and one person with MS. While four clusters may be adequate for assessing healthy tissue, in MS the extra clusters help to better distinguish tissue abnormalities.

To fully leverage the presence of the three metrics, it was important that the metrics each contributed information, as seen inFigure 2. However, having some similarity in metrics between subjects was also important. If the three metrics did not bear any similarities across subjects, the clustering would not have been as reliable upon testing.Figure S3shows metric “atlases” made by averaging over the healthy population, which shows that while MWF does vary naturally between subjects (Dvorak et al., 2021), µFA and C_MD_show less variation, thereby providing common features between subjects which was important for reliable clustering. Another alternative clustering approach only considering measures in white matter, where measures are the most consistent, is presented inFigure S4. Although this provides reasonably detailed classifications in healthy tissue, as lesions in MS were often classified as grey matter-like, including grey matter in the clustering may also be of value to help with lesion delineation. While cluster number changes leading from more to less myelinated tissue are presented inFigure 6in the difference maps, the opposite cluster number changes are also important to evaluate, and are presented inFigure S5. Opposite cluster number changes (going from less to more myelinated) were largely present at borders of tissue classes, suggesting that these may be related to interpolation and partial volume effects, while cluster changes from more to less myelinated were present in large portions of tissue, more likely to be true microstructural changes.

More metrics may have provided more detailed tissue classifications at the expense of higher data dimensionality and false cluster numbers, while fewer metrics would have been more biased by the drawbacks of the acquisition itself. The similar cluster sizes may be a by-product of the chosen clustering algorithm; different algorithms may provide more dissimilar cluster sizes at the expense of less reliable tissue classes. Similar approaches have been previously presented using only diffusion metrics for glioma gradation as inInano et al. (2014), or using quantitative T_1_and T_2_values to segment thalamic nuclei as inDeoni et al. (2007). Diffusion–relaxation correlation studies also aim to use multidimensional data to categorise tissue, but combine diffusion- and relaxation-based measurements into the same acquisition scheme to resolve intra-voxel tissue properties and create maps of pre-defined tissue characteristics (Martin et al., 2021;Narvaez et al., 2022).

### 4.2 Clusters in MS

At a group level, normalised sizes of clusters varied significantly in MS compared with HCs. When comparing cluster sizes, general trends of change were observed between HC, RRMS, and ProgMS. Significantly lower proportions of Cluster #1 (with characteristics of tissue in regions of highly coherently aligned white matter) and increased proportions of Cluster #4 (with characteristics of tissue in sub-cortical white/grey matter) and Cluster #6 (with characteristics of tissue in iron-rich deep grey matter) showed generally decreased myelin content, increased axonal injury, or loss and increased iron concentration in MS. Cluster #4 differs from Cluster #3 primarily due to decreased anisotropy and higher heterogeneity, which in the context of MS indicates not only demyelination which is present in #3 as well, but also increased damage to axons and heterogeneity. The excess of Cluster #4 in MS compared with HC, particularly in ProgMS, could be attributed to an increase in cellularity (more glial cells), reflecting an increase in microglia activity throughout the brain, which is associated with disease progression due to a smouldering disease process of ongoing chronic inflammation (Pukoli & Vécsei, 2023). Previous studies found generally higher levels of axonal damage and loss than myelin loss in MS tissue (Rahmanzadeh et al., 2021;Shirani et al., 2019;Wang et al., 2011), which may be reflected here by the presence of higher amounts of Cluster #4. A multimodal “signature” for astrogliosis-induced scarring (Correale, 2014) at the grey–white matter boundary is marked by increased T_2_relaxation times and increased mean diffusivity due to looser axonal packing resulting from the presence of astrocytes (Benjamini et al., 2021,^2023^). In our work, the microstructural features of Cluster #4 correspond with the signature of increased T_2_times and mean diffusivity, and Cluster #4 was largely present at the grey–white matter border, suggesting that this cluster might also encompass such scarring, which would have mixed cell density and loose axonal packing.

Lesions were generally classified as Cluster #3, Cluster #4, or a mix of both. This suggests differences in lesion microstructure, with Cluster #3 likely representing lesions with demyelination and some axonal damage, Cluster #4 representing lesions with demyelination, axonal loss and edema, and lesions with both classes representing regions of demyelination, axonal loss and damage, edema, and inflammation. One histology-defined lesion classification system defines lesions as active, inactive, and mixed active/inactive (Kuhlmann et al., 2017). Active lesions have been characterised as showing demyelination and hypercellularity with dense infiltration of microglia and axonal loss, inactive lesions are thought to be hypocellular with some axonal damage and demyelination, and mixed active/inactive lesions show an inactive core surrounded by active microglia (Kuhlmann et al., 2017). In the present work, the interpretation for Cluster #4 lesions corresponds roughly with active lesions, Cluster #3 with inactive, and Cluster #3 and Cluster #4 mixed lesions as mixed active/inactive lesions. The acquired resolution (3 x 3 x 3 mm^3^) of µFA and C_MD_images likely did not allow for clear distinction of an inactive core and active rim in such cases; higher resolution MRI acquisitions may allow for better lesion delineation.

The increased proportion of Cluster #6 in MS is also of note. While Cluster #6, driven primarily by high MWF, was interpreted as iron-heavy due to its presence in deep grey matter regions, its increase in MS may also be in part due to other features of the MWF. MWF can be heightened by high iron concentration in blood vessels, and may be artificially raised due to ventricle pulsation resulting in small changes to ventricle size (Abderezaei et al., 2021;Feinberg & Mark, 1987;Sloots et al., 2020;Zhu et al., 2006), further exacerbated by spatial regularisation in the MWF calculation. In MS, Cluster #6 may be more prevalent around ventricles due to atrophy and subsequently more noticeable ventricle pulsation artifacts. The increase in #6 just around ventricles may also be due to small errors in registration and interpolation; for example, if µFA and C_MD_have low values just around the ventricles due to partial voluming and interpolation, and MWF is raised due to ventricle pulsation artifacts, this may count as Cluster #6. However, changes in MS from Clusters #1 to #6 (5-cluster changes in difference maps, from highly ordered myelinated deep white matter to iron-rich tissue) are not typically seen in these peri-ventricular areas, and potentially do constitute high iron regions.

### 4.3 Severity scores

Severity scores highlight changes to white matter microstructure in MS. A cluster difference of 1 shows a changing white matter gradation from more myelinated to less myelinated, with decreasing anisotropy and increasing heterogeneity. Severity scores in MS, therefore, represent a trend towards demyelination, axonal damage/loss, and inflammation in white matter, with higher severity scores indicating more extensive areas of change.

Significant correlations between severity scores and SDMT were found in all MS, but particularly in ProgMS, echoing findings from previous studies which linked subtle changes in NAWM to cognitive ability (Abel et al., 2020;Dineen et al., 2009;Pokryszko-Dragan et al., 2018). These previous works identify changes in myelin content and edema in specific brain structures, but correlations were weaker than those found in the current study. Looking at changes in myelin content, axonal integrity and tissue heterogeneity simultaneously results in a substantially stronger relationship with SDMT scores, which suggests that a combination of all these changes in white matter drives cognitive decline.

It is of note that severity scores derived from more dramatic cluster changes such as 2-cluster (Spearman’s r = 0.259, P = 0.221 for all MS), 3-cluster (Spearman’s r = 0.103, P = 0.660 for all MS), or higher changes did not correlate significantly with SDMT scores, suggesting that a likely driver of poorer cognitive performance is the subtle change in NAWM integrity, rather than more dramatic changes to microstructure such as lesions. Severity scores did not correlate significantly with EDSS, which is primarily related to mobility; investigating specifically motor tracts and the spinal cord may provide stronger relationships with EDSS.

### 4.4 Limitations

There are some limitations to consider in this study. Care must be taken not to over-interpret the meaning of each cluster and changes to tissue classifications, as each cluster is a function of three different metrics. Interpolation and partial volume effects may have influenced the classification, as the µFA and C_MD_maps were acquired at a relatively low resolution and registered to the higher-resolution MWF maps. This may have particularly impacted clusters immediately next to the ventricles, and at the grey–white matter boundary. Our diffusion data included partial Fourier and were reconstructed on the Philips scanner using an antisymmetric filter, which mitigates some of the drawbacks of partial Fourier reconstruction with regard to Gibbs ringing (McGibney et al., 1993). However, the Gibbs ringing removal performed as part of data processing could still be improved as inLee et al. (2021). As the derived maps were also then interpolated to a higher resolution, partial Fourier may not have had a large impact on clusters.

Templates and metric atlases were created based on the 25 HCs, and registration of the resulting atlas of tissue classification to each MS participant and the creation of difference maps may have been biased simply by differences between HC and MS brain. For example, MS-related atrophy could result in some mis-registrations. However, at the image resolutions used here, the effect is quite minor. Group-level analyses comparing MS with HCs may include the effect of atrophy; we aimed to mitigate this by warping atlases to MS and CSF masking the atlas based on the MS CSF mask, and also allowing individual analyses as inFigure 6. A study-specific atlas could be created based on the population of interest (e.g. MS), which would improve some potential mis-registrations, but this would limit generalisability; instead, we focused on creating a HC atlas which can continue to be built up to include a wide range of ages, head sizes, etc., and used as a universal reference. In future, templates including neurological conditions may improve registration effects. With very large datasets or meta-analyses, MS phenotype-specific atlases may help elucidate the difference in tissue classification compared with a healthy atlas, including variations in specific brain locations, at a group level.

The atlas of tissue classification included participants with a broad age range, and as MWF and measures from diffusion imaging are known to vary with age (Billiet et al., 2015;Dvorak et al., 2021), this may have influenced the results. However, age has a less pronounced impact than that of disease itself, making the results found here still valid, although care must be taken not to neglect the impact of age when modelling with larger datasets. Age-specific atlases may be made with bigger datasets and become more accurate targets for age-matched comparison with disease. Region-specific atlases may also highlight localised changes to tissue microstructure that may drive neurological conditions.

## 5 Conclusion

In this work, we present a flexible, microstructure-based tissue classification framework and demonstrate its use in MS to map classifications of damaged tissue. The healthy atlas of tissue classification can act as a comparison tool for other datasets using the same MRI acquisitions. Tissue damage maps showed regions of microstructural tissue damage not visible on conventional images. Associated severity scores, which come directly from underlying microstructural features of white matter, were found to be related to cognitive ability in MS. This format of clustering purely quantitative data from multiple MRI techniques to provide “microstructure maps” can be extended to more and other metrics and clustering algorithms. Applications could include not only other neurological diseases, but also other body parts provided the metrics follow the rough guidelines of non-overlapping information and similarity between subjects.

## Supplementary Material

Supplementary Material

## Data Availability

Clustering models and the atlas of tissue classification are available online athttps://zenodo.org/records/11399015, with code to generate the same available athttps://github.com/sharadab/caqe.
