# Supplementary material for: Microstructure-informed brain tissue classification using clustering of quantitative MRI measures
Source: Imaging Neurosci (Camb). 2025 Apr 3;3:imag_a_00526. doi: 10.1162/imag_a_00526 (PMC12319851; doi:10.1162/imag_a_00526)
Supplement: Supplementary Material [file imag_a_00526-supp.pdf]

Supplementary Materials for:

Microstructure-informed brain tissue classification using clustering of quantitative MRI measures

Sharada Balaji*, Marek Obajtek, Irene M. Vavasour, Adam Dvorak, Guillaume Gilbert, Poljanka Johnson, Roger Tam, Cornelia Laule, David K.B. Li, Anthony Traboulsee, Alex MacKay, Shannon Kolind
*Corresponding author: Sharada Balaji (sbalaji@phas.ubc.ca)

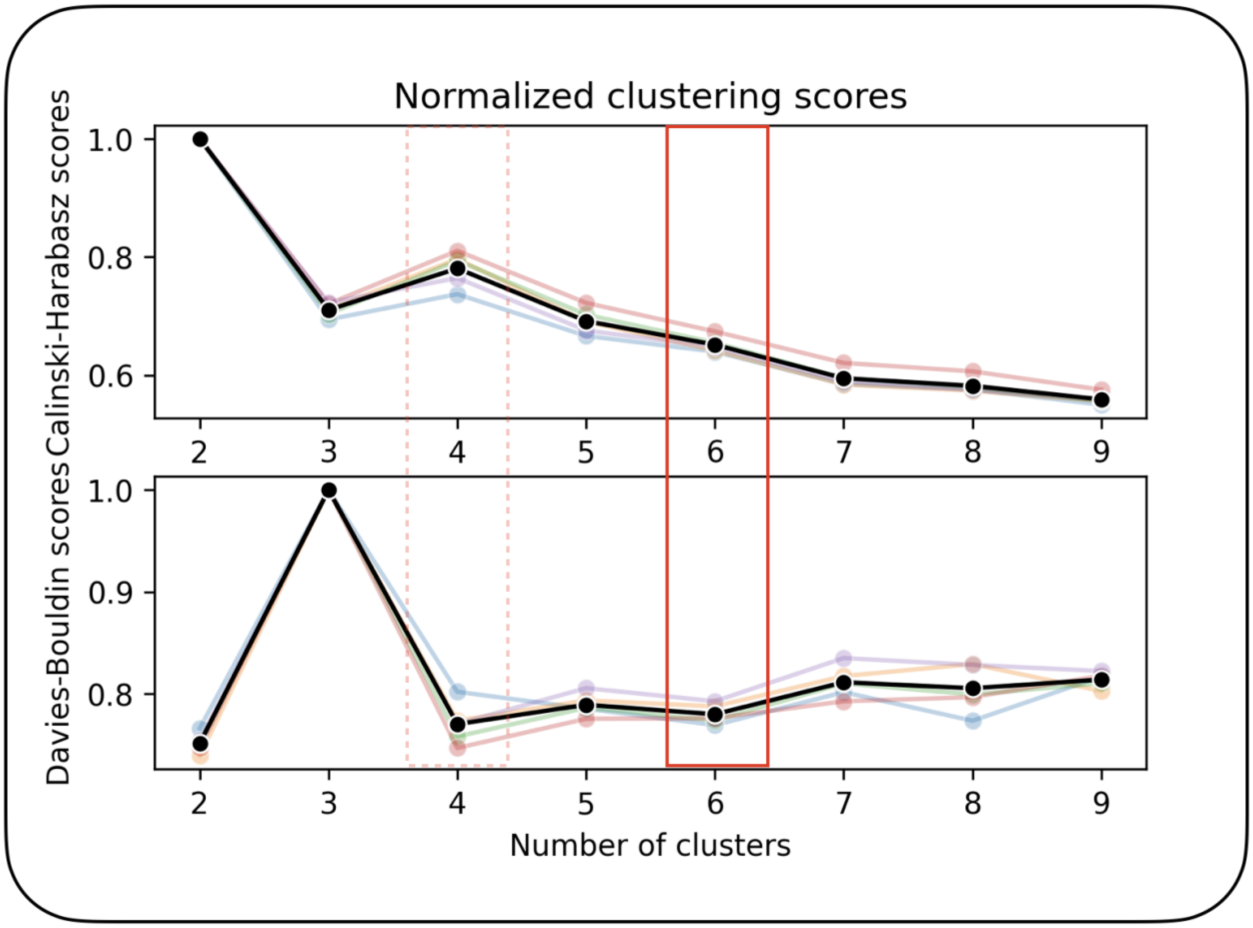

Fig. S1. Clustering scores from a representative set of 5 healthy test subjects. Subjects were classified based on clustering with a separate 20 healthy training subjects. Higher Calinski-Harabasz scores and lower Davies-Bouldin scores are desirable. Solid red boxes indicate the chosen cluster number, and dashed red boxes represent potential other "good" cluster numbers. Different coloured lines represent different subjects, while the black lines represent the average of 5 subjects. While 4 and 6 clusters were viable options, 6 was chosen as the best number after looking at several classified examples for most consistency between subjects and most detailed tissue segmentation without over-detailing.

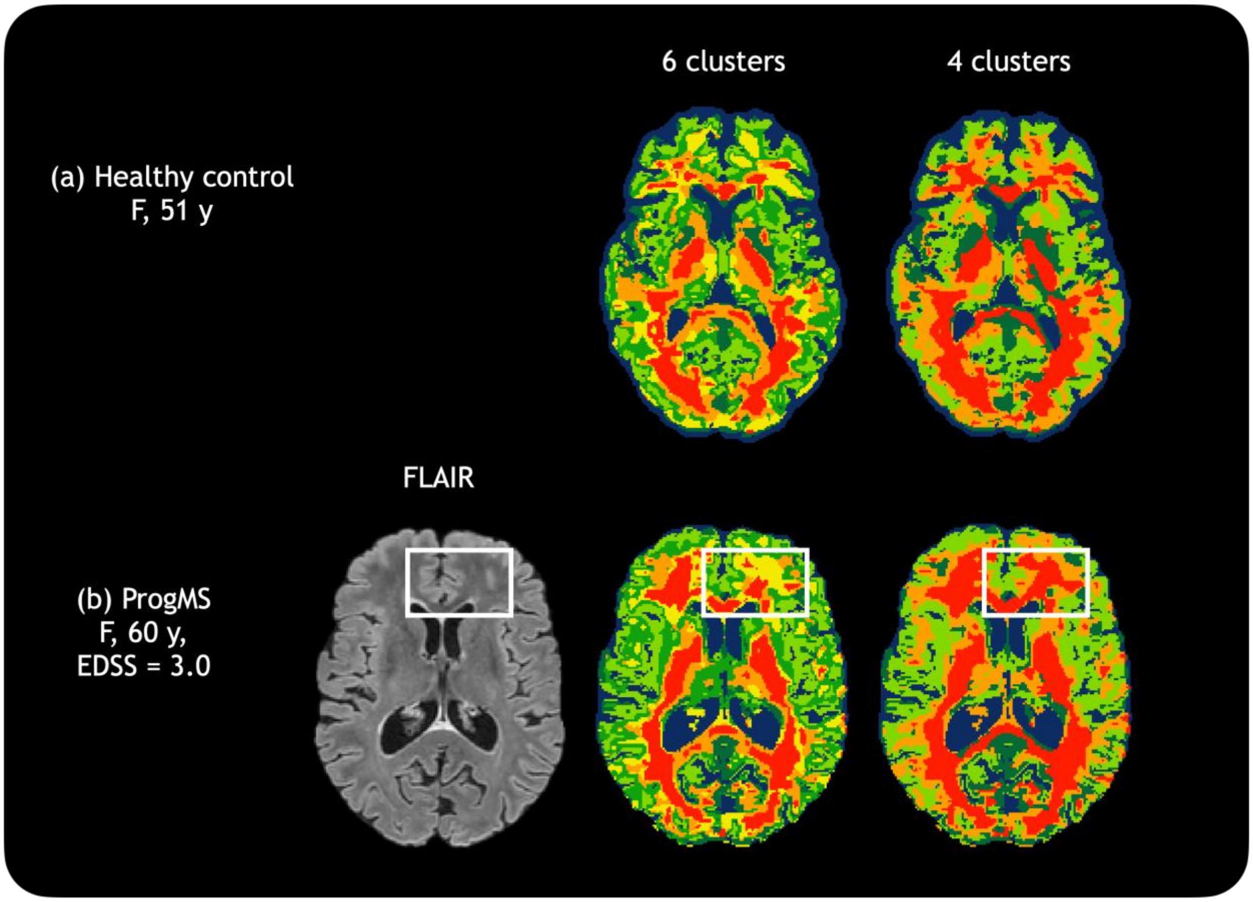

Fig S2. 6-cluster and 4-cluster tissue classification. Comparison of the 6-cluster tissue classification presented in the main paper and a 4-cluster classification in (a) one healthy control and (b) one person with MS. The classification derived from 4 clusters broadly fell into regions of deep white matter, sub-cortical white matter, cortical grey matter, and iron-rich tissue. While this may be adequate in HCs, the advantage of 6 clusters becomes clearer in MS: in (b), the white box marks a region that is classified in more detail as a region of lower myelination and anisotropy by the 6-cluster classification, while the 4-cluster classification does not show major differences.

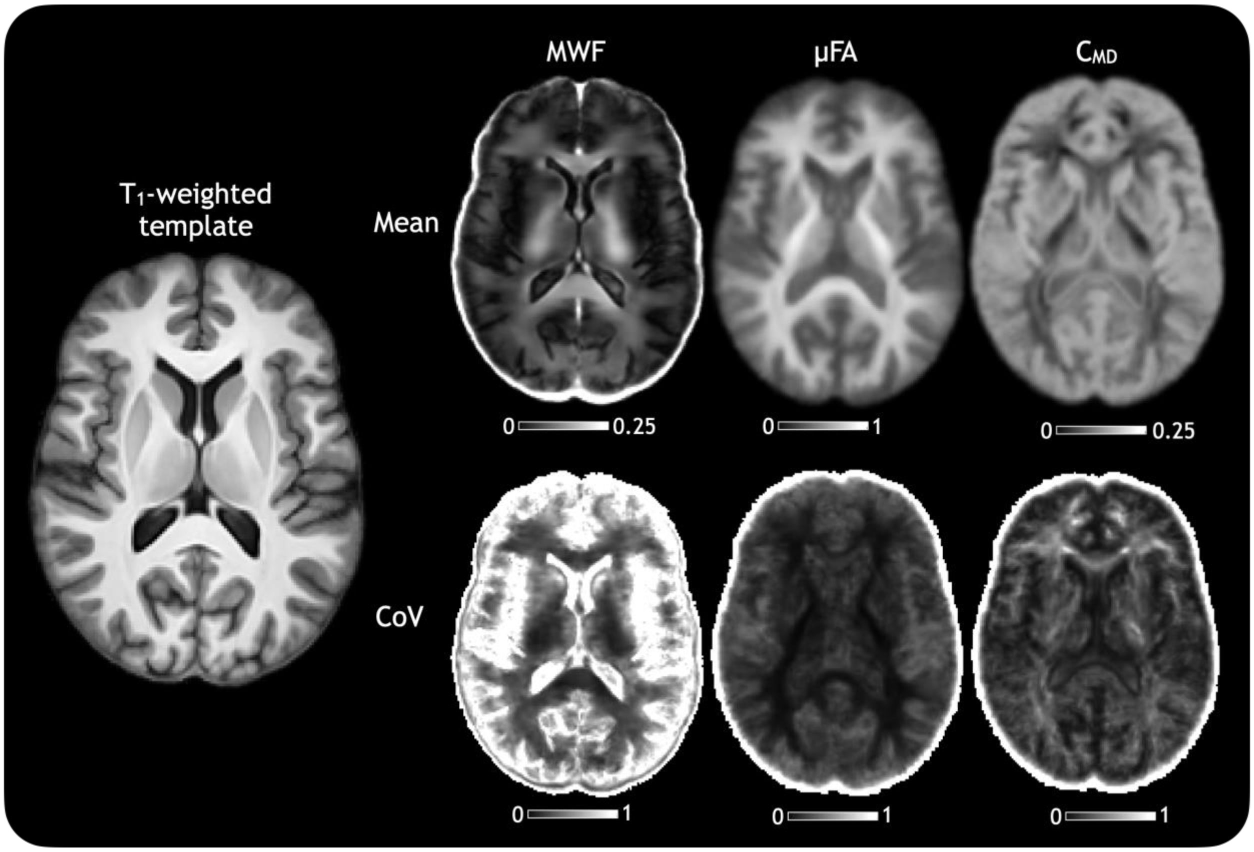

Fig. S3. Population-averaged "atlases" of MWF, μFA and C_{MD}. Atlases were made from the healthy population spanning an age range of 23-70 years (mean=46 years). From the coefficient of variation (CoV = Standard deviation/Mean) maps, MWF shows the most variation between subjects, followed by C_{MD} and then μFA. As μFA shows the least variation between subjects, it was useful for providing consistency in clustering (for example, for showing regions of highly coherent white matter as the same cluster across all test subjects even when classified based on a different training set).

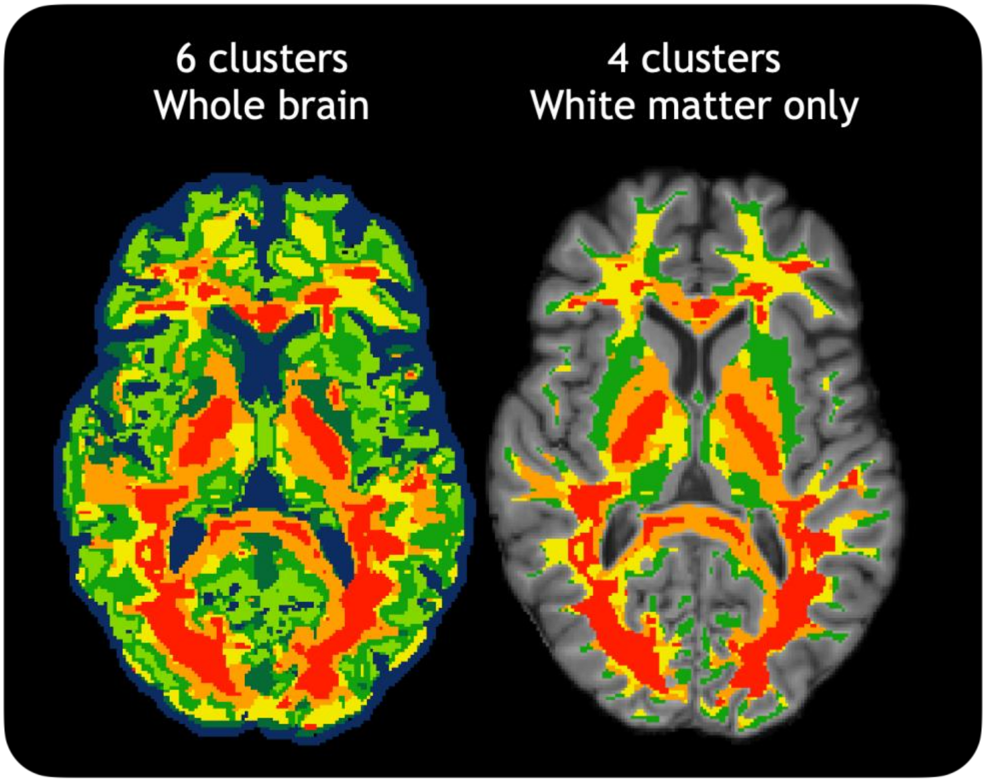

Fig S4. Considering only white matter. As white matter was the primary focus of the paper and measures show lower CoV in white matter, an alternative clustering using only white matter was attempted. White matter was preferentially clustered into 4 classes based on clustering 20 HCs and testing on 5 HCs, perhaps leading to over-detailing. Shown here is an example in a healthy control. The advantage of clustering whole brain can be better appreciated in MS: in the 6-cluster classification of MS tissue, as lesions were often categorized as sub-cortical white matter and sub-cortical grey/white matter, it is important to also cluster grey matter and allow better delineation of lesion types.

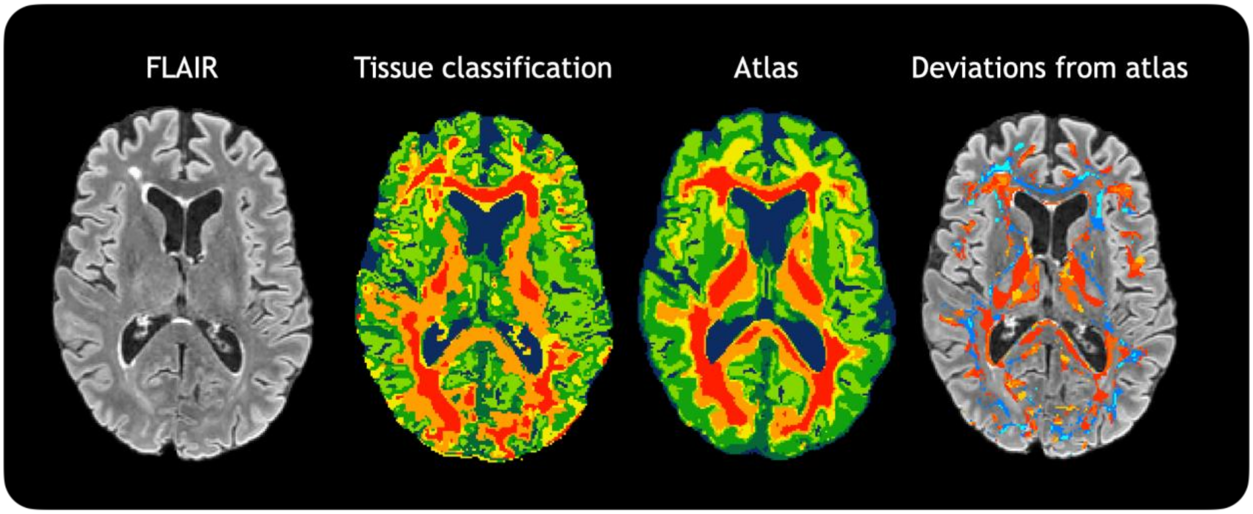

Fig S5. Both types of cluster changes. Comparison of deviations from the atlas in a person with MS that are not only representative of demyelination (shades of red), but also of increased myelin content (shades of blue). Over all MS participants, the regions of increased myelin content (blue) constituted 6.1% of voxels while the demyelination (red) constituted 13.6%. It is also of note that the blue voxels generally fell at the borders of tissue categories, suggesting that they capture interpolation and partial volume effects rather than true pathology, while red voxels fell in larger parcels of tissue, more likely representing real changes to tissue.
